# Editorial: Neuroinflammation and cognitive impairment

**DOI:** 10.3389/fnagi.2024.1453772

**Published:** 2024-07-09

**Authors:** Juan Li, Yang Wang, Kun Xiong, Chengjin Gao

**Affiliations:** ^1^Emergency Department, Xinhua Hospital Affiliated to Shanghai Jiao Tong University School of Medicine, Shanghai, China; ^2^Department of Neurosurgery, Shanghai Ninth People's Hospital Affiliated to Shanghai Jiao Tong University School of Medicine, Shanghai, China; ^3^Department of Anatomy and Neurobiology, School of Basic Medical Science, Central South University, Changsha, Hunan, China

**Keywords:** neuron damage, neuroinflammation, cognitive impairment, microglia, astrocyte

The relationship between neuroinflammation and cognitive impairment represents a crucial area of contemporary neuroscience research. Neuroinflammation pertains to the inflammatory response within the central nervous system (CNS), typically initiated by the activation of immune-related cells like microglia and astrocytes (Figure 1). This inflammatory response is believed to play a significant role in various neurodegenerative diseases and cognitive impairments (Chen et al., 2024). Understanding the specific mechanisms and effects of neuroinflammation can aid in the development of new treatment methods aimed at alleviating or preventing cognitive decline associated with it (Ban et al., 2024).

**Figure 1:**
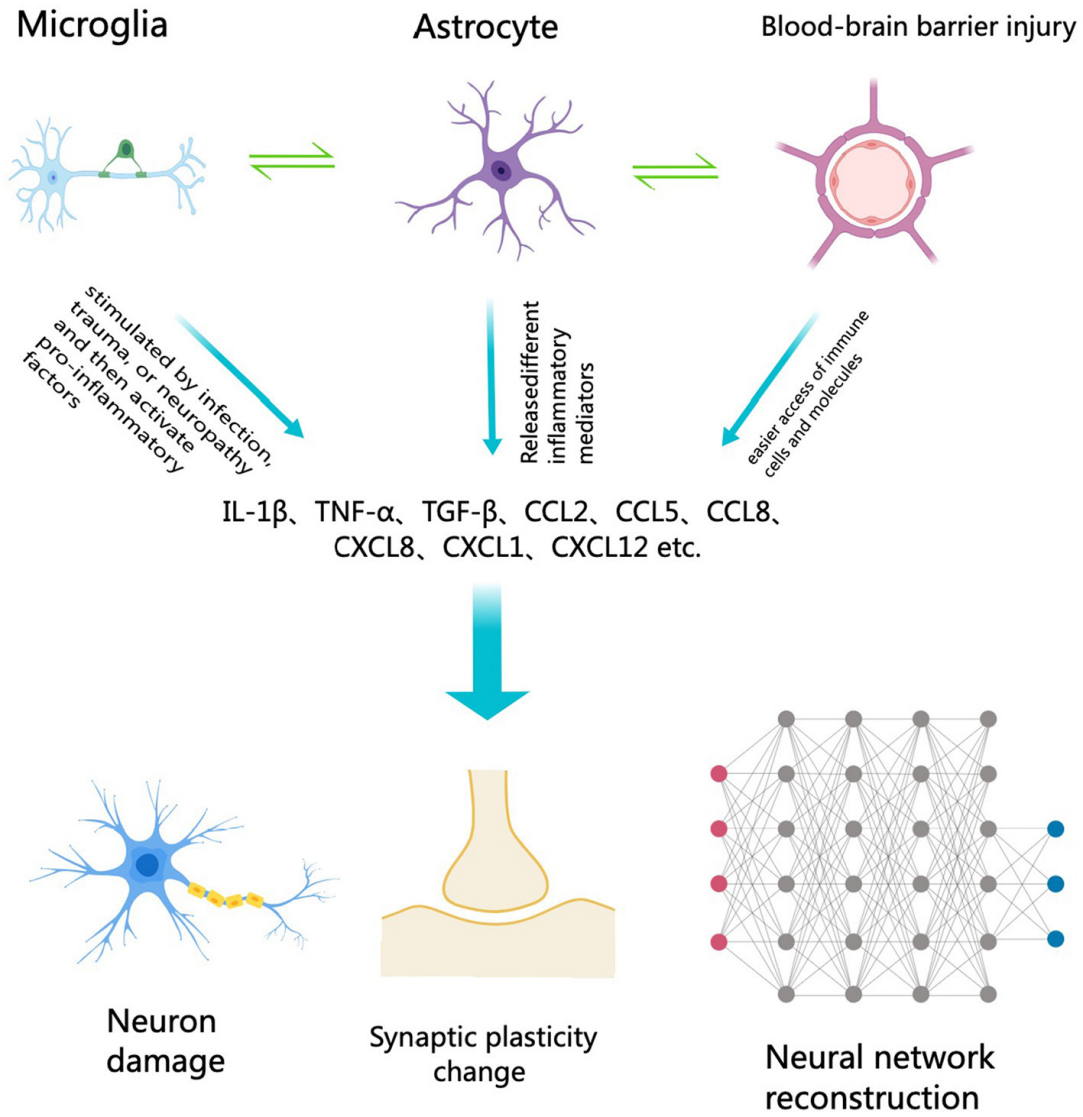
The relationship of neuroinflammation and cognitive impairment.

Tan et al. suggested that peripheral inflammation may be an important cause of cognitive dysfunction in the CNS. The author systematically expounded this problem from the aspects of the correlation between peripheral inflammation and cognitive dysfunction, the affected brain regions and functional networks, and the mechanism of peripheral inflammation inducing central inflammatory response. The primary mechanism through which peripheral inflammation promotes central inflammatory immune activation involves the facilitation of peripheral immune cell infiltration into the CNS and subsequent activation of central innate immune cells, thereby contributing to the development of neurocognitive impairment. In addition, severe systemic inflammation due to infection can also cause damage to the nervous system through excessive production of inflammatory mediators, including complement factors, prostaglandins, cytokines, and chemokines. The authors proposed a preventive and therapeutic strategy based on the peripheral organ-brain physiological axis, which provides a theoretical framework for further research and application.

Inflammatory pain is one of the most prevalent and difficult-to-treat human conditions, where peripheral and central sensitization may influence its chronicity and treatment resistance. Wu et al. analyzed the potential mechanisms through which exercise alleviates inflammatory pain. These mechanisms include the regulation of synaptic plasticity in the anterior cingulate cortex, modulation of the endocannabinoid system, adjustment of excitatory balance in the dorsal horn of the spinal cord, regulation of immune cell polarization, modulation of cytokine levels, and control of glial cell activity. The experimental results demonstrate that exercise effectively lowers the levels of inflammatory factors, reduces pain sensitivity, enhances behavioral pain responses, and exerts anti-inflammatory and analgesic effects.

Systemic immune disorders can lead to cognitive dysfunction (Zhao et al., 2022; Zang et al., 2023). Current studies have shown that diet is an important way to regulate the immune system, and some dietary nutrients and dietary patterns have shown potential to regulate inflammatory states (Christ et al., 2019; Mcgrattan et al., 2019; Iddir et al., 2020). The Dietary Inflammation Index (DII) has been widely used to assess the ability of dietary patterns to influence inflammatory status and to assess the association between diet-induced inflammation and various diseases (Shakya et al., 2021; Hariharan et al., 2022). Zhang, Peng et al. conducted a cross-sectional study to investigate the relationship between DII and cognitive function and found that DII scores were significantly positively associated with low cognitive function on Animal Fluency test and Digit Symbol Substitution Test assessments. Smooth curve fitting results showed that there was a non-linear relationship between DII and Digit Symbol Substitution Test assessment of cognitive decline. Subgroup analysis revealed that individuals over 75 years old and women were more likely to show an association between high DII scores and cognitive impairment.

Postoperative cognitive dysfunction (POCD) is a prevalent neurological complication in elderly patients following general anesthesia or surgery. It is characterized by cognitive decline that can persist for weeks, months, or even longer (Feinkohl et al., 2023). Electroacupuncture therapy (EA), which combines physical nerve stimulation and traditional Chinese acupuncture treatment, is expected to be a potential treatment for the prevention and treatment of POCD (Ou et al., 2021; Sun et al., 2022). While the benefits of EA on POCDs have been explored in preclinical and clinical studies, the reliability of EA is limited by methodological deficiencies and the underlying mechanisms have not been fully elucidated. Zhao and Zou explored the potential mechanism of EA to improve POCD by analyzing the effects of EA on neuroinflammation, oxidative stress, autophagy, gut-brain axis and epigenetic modification. The results indicate that EA can protect nerve function through several mechanisms: inhibiting inflammatory factors and microglial cell activation, enhancing antioxidant defense, regulating autophagy, improving intestinal microecological balance, and modulating the expression of specific microRNAs.

Phosphatase and Tensin Homolog is an important tumor suppressor, which can inhibit tumor cell survival, proliferation and energy metabolism (Shan et al., 2019). Highly expressed in neurons, it plays an important role in neurogenesis, synaptogenesis, and neuronal survival, and loss of its activity can also lead to abnormal neuronal function and has been associated with a variety of neurological disorders, including stroke, seizures, and autism (Igarashi et al., 2018; Pan et al., 2022; Zheng et al., 2023). Through literary metrology analysis, Zhang, Tan et al. discovered that the Phosphatase and Tensin Homolog/Phosphoinositide 3-kinase/Protein kinase B pathway can regulate protein synthesis. Furthermore, this pathway has implications in synaptic enhancement, neurite growth, and the specification and regeneration of axons and dendrites by modulating Bcl-2-associated death promoter/B-cell lymphoma 2.

Ischemic stroke is a leading cause of death and disability worldwide and can lead to increased neuroinflammation, neurological impairment and cognitive impairment after stroke (Alsbrook et al., 2023; El Husseini et al., 2023). Song et al. found that knocking out receptor-interacting protein kinase 1 (Ripk1) and N-ethylmaleimide-sensitive fusion ATPase (NSF) resulted in a reduction in infarct and edema volume, accompanied by an improvement in neural deficits in the transient middle cerebral artery occlusion mouse model. Ripk1 and NSF play a crucial neuroprotective role in ischemic stroke by modulating the Receptor-interacting protein kinase 1/Receptor-interacting protein kinase 3/Mixed Lineage Kinase Domain-Like signaling pathway associated with neuronal necrotic apoptosis. Knockdown of Ripk1 and NSF serves as a promising neuroprotective strategy to mitigate brain damage and enhance neurological recovery following ischemic stroke.

Neuroinflammatory processes play a crucial role in the pathophysiology of various neurodegenerative diseases, including idiopathic atmospheric hydrocephalus (iNPH) (Rauf et al., 2022). Through meta-analysis, Zhao and Zou identified that Interleukin-16, Urokinase-type plasminogen activator, and Urokinase-type plasminogen activator play roles in the pathogenesis of iNPH. Moreover, iNPH may influence the expression of Glial cell line derived neutrophic factor, Matrix metalloproteinase-1, and Interleukin-12p70. Consequently, targeting these specific inflammatory markers could represent a prospective strategy for the treatment and prevention of iNPH.

The aforementioned studies offer diverse perspectives on investigating the impact of neuroinflammatory responses on cognitive impairment. Given the current research focus and urgency in this area, we anticipate that this topic will capture the interest of researchers across various disciplines, including basic neuroscience researchers and neuroclinical experts. This research theme promises to contribute cutting-edge insights into the advancement of this field, particularly in understanding the interplay between neuroinflammation and cognitive decline. The objective is to stimulate the publication of original research findings and offer innovative perspectives.

## Author contributions

JL: Writing – original draft. YW: Supervision, Writing – review & editing. KX: Conceptualization, Writing – review & editing. CG: Supervision, Writing – review & editing.
